# Using RAFT Polymerization Methodologies to Create Branched and Nanogel-Type Copolymers

**DOI:** 10.3390/ma17091947

**Published:** 2024-04-23

**Authors:** Athanasios Skandalis, Theodore Sentoukas, Dimitrios Selianitis, Anastasia Balafouti, Stergios Pispas

**Affiliations:** 1 Theoretical and Physical Chemistry Institute, National Hellenic Research Foundation, 48 Vassileos Constantinou Avenue, 11635 Athens, Greece; dimitrissel404@gmail.com (D.S.); anastasia.mplft@gmail.com (A.B.); 2 Centre of Polymer and Carbon Materials, Polish Academy of Sciences, 34 M. Curie-Sklodowska Street, 41-819 Zabrze, Poland

**Keywords:** branched, copolymers, RAFT polymerization, stars, nanogels, grafts, hyperbranched

## Abstract

This review aims to highlight the most recent advances in the field of the synthesis of branched copolymers and nanogels using reversible addition-fragmentation chain transfer (RAFT) polymerization. RAFT polymerization is a reversible deactivation radical polymerization technique (RDRP) that has gained tremendous attention due to its versatility, compatibility with a plethora of functional monomers, and mild polymerization conditions. These parameters lead to final polymers with good control over the molar mass and narrow molar mass distributions. Branched polymers can be defined as the incorporation of secondary polymer chains to a primary backbone, resulting in a wide range of complex macromolecular architectures, like star-shaped, graft, and hyperbranched polymers and nanogels. These subcategories will be discussed in detail in this review in terms of synthesis routes and properties, mainly in solutions.

## 1. Introduction

The recent development of controlled radical polymerization techniques has driven researchers to synthesize more complex and precise architectures with different monomers over the past 50 years utilizing several methods, such as atom transfer radical polymerization (ATRP) [1], ring-opening polymerization (ROP) [2], nitroxide-mediated polymerization (NMP) [3], and reversible addition-fragmentation chain transfer polymerization (RAFT) [4]. Copolymerization of two or more different monomers has a distinct advantage over conventional homopolymers since the synthesized copolymers incorporate the properties of distinct monomers or parent homopolymers and can be amphiphilic or double-hydrophilic exhibiting multiple functionalities. When dispersed in aqueous media, copolymers self-assemble in different structures, such as micelles [5], vesicles [6,7], worms [8], etc., depending on their amphiphilicity, degree of polymerization, and architecture. In this way, they can act as nano reservoirs to encapsulate hydrophobic molecules [9] and/or complexes with hydrophilic (bio)macromolecules [10,11]. Careful choice of monomers can also lead to environmentally stimuli-responsive structures that can be further utilized for smart delivery and release of therapeutic molecules [12,13,14,15].

Branched polymers are a special category incorporating structures comprising several side chains/branches covalently attached to a main chain/backbone. Two or more polymers can be utilized to synthesize branched polymers, while a mixture of synthetic and natural polymers is also possible [16,17]. Grafts, stars, hyperbranched, and nanogels can be included in this category. Graft copolymers are a subcategory of branched polymers with a distinct long main chain/backbone linked covalently with several side chains. Grafting can be achieved by “grafting through,” “grafting onto,” and “grafting from” synthesis routes depending on the monomers and desired final architecture [18]. Star copolymers are macromolecular architectures comprising a core domain and extended arms/chains (of homopolymer or copolymer nature). The synthetic route for obtaining them can be either a “core-first” or “arm-first” methodology [19]. Hyperbranched copolymers are highly branched, or dendritic-like structures comprised of monomers bearing multiple terminal units and are primarily determined in terms of macromolecular structure and properties by the degree of branching [20]. Nanogels are physically or chemically crosslinked hydrogel-like nanostructures [21,22]. In the chemically crosslinked case, the nanogel can be regarded as a single macromolecule with nanoscale dimensions and an intrinsic three-dimensional branched structure.

Compared to other structures, branched polymers have shown increased loading capacity of therapeutic molecules and other solvent non-soluble species (e.g., drugs, dyes, metal nanoparticles, etc.). Due to their branching density, the amphiphilic nature, and the abundance of different functional groups, branched polymers can be utilized in several aspects of biomedical applications. Another characteristic is their tunability in every aspect concerning branching degree, degree of polymerization, the nature and size of backbone and side chains, and the amphiphilicity ratio that can, in turn, improve specific abilities, change self-assembly properties, decrease biotoxicity, and increase the loading/complexation capacity. Up-to-date studies reveal the strong and multifaceted potential of branched copolymers for biomedical applications in drug [23], gene [24,25], and tissue engineering [22,26].

RAFT polymerization is a versatile polymerization method used with various monomers, solvents, and at a broad temperature range. A chain transfer agent (CTA) is a characteristic molecule which mediates RAFT polymerization. RAFT agents typically bear a thiocarbobylthio group that comprises a Z- and an R-group, the careful choice of which is crucial for maintaining the radical equilibrium during polymerization and the control of the final polymers and polymeric nanostructures [4,27,28,29]. RAFT polymerization has several interesting variations, such as the polymerization-induced self-assembly (PISA), which is mediated most frequently in water; the forming copolymers are simultaneously self-assembled in the course of the polymerization process in various nanostructures, such as spherical micelles, worms, vesicles, etc. [30,31,32,33]. RAFT-mediated photopolymerization is another variation of the method, utilizing a photosensitizer as the initiator, where different wavelengths can activate specific chain transfer agents to further mediate the polymerization [30,34,35,36].

This review deals with the most recent studies regarding branched polymers, the limitations and advances in their synthesis and characterization, as well as their properties, potential applications, and future perspectives.

## 2. RAFT Polymerization

RAFT (reversible addition-fragmentation chain transfer) polymerization is a controlled/living radical polymerization technique that allows for precise control over the polymer architecture, molecular weight, and polydispersity. Developed in the late 1990s, this method has gained prominence for its versatility and efficiency in synthesizing polymers with complex structures.

The RAFT process is characterized by three main steps:

Initiation: The polymerization is initiated by radicals generated from an initiator, which react with monomers to form the initial polymer radicals.

Propagation with Chain Transfer: The growing polymer radicals react with the CTA, forming dormant species. This species can then fragment, generating a new radical that can either initiate a new polymer chain by reacting with a monomer or continue the growth of an existing chain. This controlled chain transfer mechanism is key to maintaining the living nature of polymerization.

Termination: Although RAFT is designed to minimize termination events, some termination can occur, usually at very low rates, preserving the living nature of the polymerization process.

The unique aspect of RAFT polymerization is its ability to provide control over the polymer molecular weight and distribution, enabling the production of polymers with narrow molecular weight distributions (low polydispersity index). Additionally, it is versatile in terms of the monomers it can polymerize, including styrenes, acrylates, methacrylates, and more, making it highly applicable in creating a wide range of functional polymers for fundamental research and industrial applications such as pharmaceuticals, biotechnology, and materials science.

In summary, RAFT polymerization stands out for its robustness, precision, and versatility, making it a cornerstone technique in modern polymer chemistry for designing and synthesizing advanced polymeric materials [4,27,28,29].

The core principle of RAFT polymerization revolves around the use of a chain transfer agent (CTA), which moderates the polymerization process through a reversible exchange mechanism. This mechanism involves the CTA reacting with the growing polymer radical to temporarily halt polymerization, then transferring the radical to a monomer or another polymer chain, which continues the polymerization process. This transfer process can occur multiple times during the polymerization, allowing for uniform growth of the polymer chains.

The choice of CTA can significantly influence the polymerization kinetics, molecular weight control, and polymer properties. The selection of CTA can vary based on the monomer category. It is essential to consider the compatibility of the CTA with the chosen monomer and reaction conditions, as well as the desired properties of the polymer product [37]. For acrylate and methacrylate monomers, a common category of used RAFT agents is dithioesters, such as ethyl cyanovaleric trithiocarbonate (ECT) or benzyl dithiobenzoate (BDB). For vinyl monomers like styrene or vinyl acetate, trithiocarbonates such as benzyl trithiocarbonate (BzTC) or isopropyl trithiocarbonate (IPrTC) are often used. They enable controlled polymerization and can yield polymers with desired characteristics. In the case of acrylamide-based monomers, trithiocarbonates or trithiocarbamates can be effective CTAs. Examples include dithiobenzoates or dithiocarbamates like dodecyl dithiobenzoate or ethyl xanthate. Similarly, for methacrylamide monomers, dithiobenzoates or dithiocarbamates are commonly employed as RAFT agents to achieve controlled polymerization. 

For monomers outside of these general categories, the choice of CTA would depend on their specific chemical structure and reactivity. Generally, dithiobenzoates, trithiocarbonates, or dithiocarbamates are versatile CTAs suitable for a wide range of monomers [29,38]. 

## 3. Graft Copolymers

Graft copolymers are another type of macromolecule with unique architecture that have drawn particular interest due to their versatility and multiple functionalities. They are composed of two parts, a main backbone and “side chains” (or grafts) covalently attached to it. Each part can be either a homopolymer, block or random copolymer, depending on the aim of the synthesis. Different functional polymeric groups can be utilized for various purposes, such as DNA complexation [39] with charged cationic groups and encapsulation of hydrophobic drugs/molecules. Recent advances in the controlled polymerization field have enabled the precise synthesis of graft copolymers with the desired architecture [18,40]. Not only that, but many researchers have used natural polymers like polysaccharides as backbones to graft synthetic polymeric side chains onto them. Introducing natural polymers and modifying their functional end groups led to synthesizing novel hybrid materials comprising both natural and synthetic counterparts. The procedure initially involves depolymerizing the polysaccharide to the desired molecular weight. As a second step, chemical modification of the functional side-groups via etherification, esterification, amination, or amidation and the introduction of a CTA makes them available for further RAFT polymerization. In this way, the polysaccharide works as a backbone, while the CTA-modified side-groups can be utilized for the grafting of additional polymer chains comprising different monomers [16,41,42,43].

**Figure 1:**
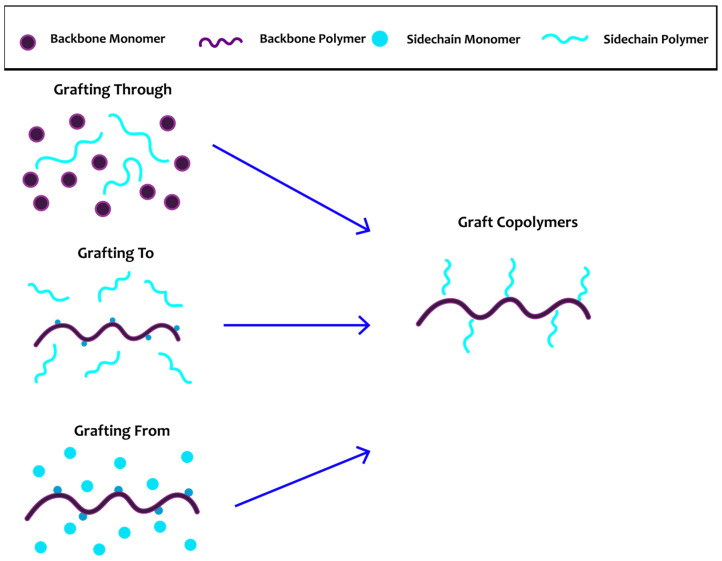
Schematic representation of grafting techniques.

Up-to-date polymer grafting is divided into three main methods: “grafting from,” “grafting to,” and “grafting through” (Figure 1). “Grafting from” uses certain side groups of the already-synthesized backbone as starting points for polymerizing the “side chains.” This method can provide excellent control and low M_w_/M_n_, resulting in well-defined copolymers with a good grafting density. “Grafting to” is the attachment of already-synthesized side chains onto a backbone, e.g., via “click chemistry.” In this way, well-defined backbone and side chains can be synthesized a priori, with the desired molecular weight and M_w_/M_n_. Although this method suffers from low grafting densities due to side reactions and steric hindrance effects, recent advances in “click chemistry” have tackled some of the issues. Lastly, “grafting through” refers to the simultaneous synthesis of the backbone and covalently attaching the already-prepared side chains. Although the side chains can be well defined and possess desired molar masses and molar mass distributions, synthesis of the backbone might initiate undesired side reactions, leading to not well-defined polymers. This review describes the most recent studies and progress made on graft copolymers in the last five years (Table 1) [16,18,43].

**Figure 2:**
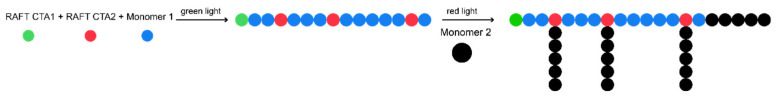
Simple schematic representation of the work of Corrigan et al. [44].

The synthesis of graft copolymers via RAFT polymerization can be achieved by utilization or modification of the R- and/or Z-groups of the RAFT CTA for preparation of the backbone and, at the same time, to create grafting points either for “click chemistry” or further polymerization with other synthetic techniques, such as ROP. As reported in the introduction, the selection of an appropriate RAFT agent is crucial for the control and yield of RAFT polymerization, as well as the final polymer architecture. In this regard, Corrigan et al. [44] have thought of a relatively simple and intelligent way for one-pot photopolymerization leading to graft copolymers. A simplified schematic representation of this work is presented in Figure 2. More specifically, they utilized two different CTA’s, 4-cyano-4-(((dodecylthio)-carbonothioyl)thio)pentanoic acid (CDTPA) and 2-(2-(n-butyltrithiocarbonate)-propionate) ethyl methacrylate (BTPEMA), selectively activated by green light irradiation but non-selective under red light irradiation. Also, BTPEMA is a methacrylate-modified CTA that can be polymerized and work as a starting point for further grafting. In this way, different graft architectures could be synthesized by selecting different irradiation wavelengths. Indeed, when the polymerization mixture was irradiated with green light, MMA and BTPEMA could polymerize “inside” CDTPA, creating a random copolymer backbone in which BTPEMA can be utilized as a starting point for further “side chain” polymerization. By enabling red light emission, poly(N, N-dimethyl acrylamide) (PDMAm) was able to polymerize both “inside” CDPTA and BTPEMA in a non-selective way, creating P((MMA-stat-BTPEMA)-block-PDMAm)-graft-PDMAm copolymers.

A similar approach was followed by Yang et al. [45], who utilized BTPEMA and 4-cyano-4-(ethylthiocarbonothioylthio)-pentanoic acid (CEPA) to prepare a multifunctional macroCTA. RAFT photopolymerization with green light only activates CEPA, so the copolymerization of BTPEMA and (ethylene glycol) methyl ether methacrylate (EGMA) leads to the formation of the backbone. The density of BTPEMA was regulated by stopping/reinitiating photopolymerization and adding more BTPEMA after intermediate PEGMA conversion. Thus, different P(EGMA-co-BTPEMA) macroCTAs were further utilized for conventional RAFT synthesis of polystyrene (PS), which was mediated via the macroCTA and the BTPEMA, leading to the synthesis of grafted copolymers in the form of P(EGMA-co-BTPEMA)-b-PS-g-PS. Further studies in aqueous dispersions show the formation of large vesicles/compound vesicles. The group also suggests this method for acrylic and acrylamide monomers since they are fully compatible with the BTPEMA RAFT agent.

Following the same pathway, Xu et al. [46] utilized oligoethylene glycol methacrylate (OEGMA) to prepare a P(OEGMA-co-BTPEMA) backbone using 2-cyano-2-propyl dodecyl trithiocarbonate (CPDTC) or CDTPA as the RAFT agent. BTPEMA was utilized in a second phase to polymerize N-isopropylacrylamide (PNIPAAm), resulting in graft copolymers with high molar mass (~100kDa), with M_w_/M_n_~1.42 and 1.47. The P((OEGMA-co-BTPEMA)-b-NIPAAm)-g-PNIPAAm copolymers present a double thermoresponsive behavior due to the presence of PNIPAAm and POEGMA. DLS studies show the formation of small nanoparticles around 20 nm in diameter, which increases to almost double when heated to 60 °C. Post-polymerization modifications include introducing pyridyl disulfide (PDS) groups that can further be utilized for crosslinking and preparation of thermoresponsive hydrogels with potential biomedical applications.

The length of both the backbone and the side chains can lead to the formation of different morphologies, while thermal and mechanical properties are also affected. Alagi et al. [47] have prepared graft copolymers comprising poly(methacrylic acid) (PMAA) backbone and poly(propylene carbonate) (PPC) side chains that were “grafted from” the carboxylic end groups. Short backbones and long side chains lead to the formation of star-like morphologies, while the opposite results in semiflexible cylinders. A longer PMAA backbone has better thermal stability and mechanical properties, though a shorter PMAA backbone can be more flexible.

Guo et al. [48] reported a combination of RAFT and ROP for the one-pot synthesis of graft copolymers. Their approach is to initially synthesize the backbone via RAFT polymerization of hydroxy monomers and continue with the ROP of ε-caprolactone from these groups. The same (4-(4-cyanopentanoic acid)dithiobenzoate) (CPADB) RAFT agent utilized for the backbone synthesis will also work as a catalyst for ROP, while the 4,4-azobis(4-cyanovaleric acid) (ACVA) initiator provides the radicals. In this manner, the group synthesized poly(hydroxyethyl methacrylate)-g-poly(ε-caprolactone) (PHEMA-g-PCL) through a one-pot procedure with very narrow molecular mass distribution and controlled molecular weights. After the successful synthesis, the group prepared brush-shaped solid polymer electrolytes via the addition of lithium perchlorate (LiClO_4_) and injection onto a polyacrylonitrile electrospinning film of 50 μm thickness. The group also reports an ionic conductivity of 4.17 × 10^−5^ S cm^−1^ at 30 °C, and lithium-ion transference number of 0.74 at 60 °C. 

Thankappan et al. [49] have prepared graft copolymers via the “grafting on” method by synthesizing the backbone and the side chains and then grafting them via “click chemistry”. The group separately prepared POEGMA-b-PMMA and PGMA via CPADB CTA, with rather low molecular mass and narrow M_w_/M_n_. Chemical modification of poly(glycidyl methacrylate) (PGMA) to PGMA-azide and alkylation of the POEGMA-b-PMMA macroCTA end group enabled the grafting of the two entities leading to the (POEGMA-b-PMMA)-g-P(GMA-N_3_), with high molecular mass of ~150 kDa and a rather good M_w_/M_n,_ around 1.3. The group also tested the self-assembly abilities of these graft copolymers in different solvent mixtures to prepare thin films.

Kim et al. [50] prepared bottlebrush- and comb-like architectures. For their synthesis, the group utilized a modified 2-(((butylthio)carbonothiolyl)thio)-propanoic acid RAFT agent by using esterification of 2-n-pentanol-2-oxazoline, an ε-caprolactone-derived inimer. This modification was further utilized for the ROP of 2-ethyl-2-oxazoline (EtOx) via the 2-n-pentanol-2-oxazoline end group and also for the conventional RAFT polymerization of N,N-dimethylacrylamide (DMA) or 2-ethylhexyl acrylate (EHA). Dispersions in aqueous media showed the formation of spherical particles around 30 nm via DLS measurements. The groups state that the nature of the graft copolymers becomes more flexible by reducing the number of CTAs on the backbone, meaning a smaller number of grafted side chains.

In the same context as the RAFT-ROP combination mentioned previously, Naguib et al. utilized a modified RAFT agent in order to prepare bottlebrush-like graft copolymers. Side chains were initially synthesized via conventional RAFT using different monomers, resulting in well-controlled molar masses and distributions. The modified RAFT macroCTA can now be further utilized to create the backbone via the “grafting-through” method by utilizing ring-opening metathesis polymerization. The final structures still maintain control over the molar mass, keeping the distributions fairly narrow for the specific combination of techniques [51].

Gegenhuber et al. combined RAFT, ROMP and “click chemistry” techniques to synthesize polymer brushes via “grafting-through”. An R-modified RAFT agent was utilized for the preparation of the PDMam side chains, which were then modified via “click chemistry”, leading to the presence of solketal-, ketoxime-, and ethoxyethyl acetal-based groups. Then, the backbone was formed via ROMP through the available groups of the R-modified RAFT agent, resulting in polymer brushes with controlled molar masses and narrow distributions [52].

Notable progress has also been made in the subcategory of polysaccharide-polymer graft copolymers via RAFT polymerization, providing precise copolymer architectures and control over the final synthesis. Assem et al. [53] prepared a CTA-modified bagasse cellulose via esterification. The cellulose-4-dimethylaminopyridine macroCTA was further utilized for the copolymerization of acrylic acid (AA) and MMA. The P(AA-co-MMA) side chains synthesized presented a very narrow M_w_/M_n_ and molecular weights of around 10 kDa. The group utilized such structures to absorb Ca^2+^ and Pb^2+^ ions from aqueous solutions, with a high success rate, especially for Ca^2+^.

Ikkene et al. [54,55] have prepared Dextran-g-poly(hydroxypropyl methacrylate) (PHPMA) via multiple chemical modifications of both Dextran and the CTA, such as oxidation, amination, and amidation, in order to combine them and prepare a backbone macroCTA. Such nanoassemblies formed spherical structures under 200 nm and exhibited increased biocompatibility compared to plain Dextran or PHPMA, making them ready for future biomedical applications. Khalid Ferji [56] has also presented an easy way to synthesize Dextran-g-PHPMA via PISA RAFT photopolymerization in UV–vis radiation. The Dextran-4-(propylthiocarbonothioylthio)-4-cyanopentanoic acid (TTC) macroCTA was prepared through simple esterification via N,N′-carbonyldiimidazole (CDI). The formed nanoassemblies had spherical shapes less than 100 nm in diameter.

Pilipenko et al. [57,58] utilized the glycosaminoglycans chondroitin sulfate (CS) and heparin (Hep) for synthesizing graft copolymers with PNIPAAm. Each polysaccharide macromolecule was converted into macroCTA using 2-(Dodecylthiocarbonothioylthio)-2-methylpropionic acid (DDMAT) and utilized further for the RAFT polymerization of PNIPAAm. The synthesized CS-g-PNIPAAm and Hep-g-PNIPAAm exhibited great biocompatibility, thermo- and pH-responsiveness in physiological media, forming spherical nanostructures of ~10 nm. The pharmaceutical potential of both systems was evaluated by loading with dexamethasone phosphate and conducting relevant studies that showed controlled release of the drug at 37 °C. The group proposed using these systems for topical, nasal, buccal, or ocular drug delivery.

## 4. Star Copolymers

**Figure 3:**
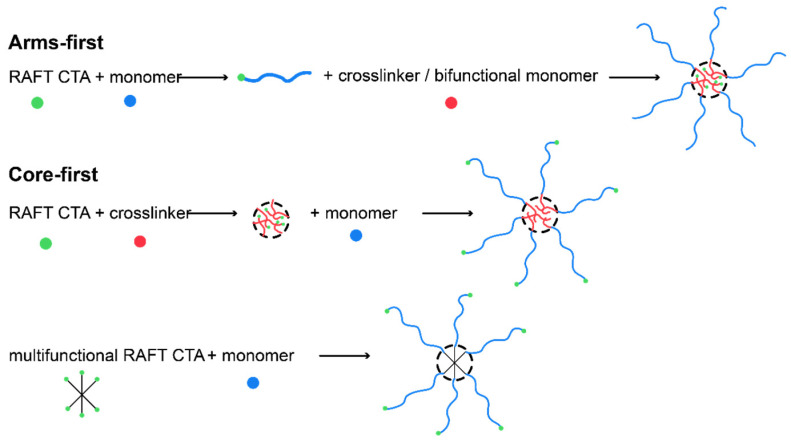
Schematic representation of star polymer synthesis routes.

Star-shaped polymers [19,59] are a notable class of polymers that belong to the branched macromolecules. Star polymers can be described as a number of linear arms linked to a central core by one of their ends (Figure 3). In this review, star-shaped polymers with linear block copolymers as arms will be examined. The vast interest in this particular macromolecular architecture is derived from the increased functionality, enhanced solution properties and lower viscosities that these materials exhibit in comparison with their linear counterparts [59]. 

There are two main synthetic routes exploited for the preparation of well-defined star-shaped polymers, namely the “arm first” [60] and the “core first” [61] approach (Figure 3). The former practically involves the preparation of linear arms and their linkage to a core (mostly small molecules like crosslinkers or coupling agents), and the latter involves the preparation of a multifunctional core and the growth of the linear arms from its active initiating sites. 

**Figure 4:**
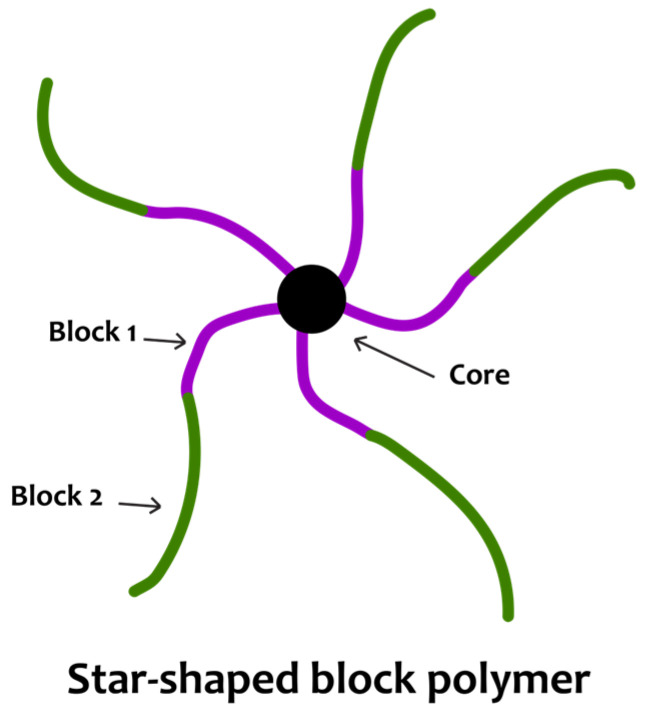
Schematic illustration of a typical star-shaped block copolymer.

The versatility of RAFT polymerization [62] has paved the road towards the evolution in the field of star-shaped block copolymers synthesis [63] as it gave researchers the ability to use a plethora of functional monomer combinations to achieve the desired properties (Figure 4). A summary of the most recent updates in the field is presented below (Table 1).

Ge and coworkers [64] investigated the preparation of star-shaped thermoplastic elastomers via RAFT polymerization. The arm-first method was used for the synthesis of the star block copolymers. Polystyrene-*b*-polyisoprene, (PS-*b*-PI) block copolymers were synthesized by RAFT and used as the arms. The crosslinked core of the stars comprises divinylbenzene (DVB) and PS. Thermal analysis by differential scanning calorimetry (DSC) and tensile testing showed that an increase in the amount of the hard segment leads to improved tensile strength, but on the other hand, it has the opposite effect on the toughness of the material.

Li Volsi et al. [65] reported on the synthesis of enzyme-degradable star polymers by RAFT polymerization to be utilized as hybrid polymethacrylate/silica inks for the preparation of 3D-printed tissue scaffolds. A collagenase cleavable peptide sequence (GLY-PRO-LEU-GLY-PRO-LYS) end-capped with dimethacryloyl groups was introduced as the crosslinked core and either poly(methyl methacrylate)/pol [3-(trimethoxysilyl)propyl methacrylate], as PMMA-*b*-TMSPMA or TMSPMA-*b*-PMMA, block copolymers as the arms. It was found that the macromolecular architecture, i.e., the sequence of TMSPMA and MMA, plays a critical role as far as parameters such as the rate of degradation, mechanical properties, and 3D printability are concerned. The copolymers in which the TMSPMA block was closer to the star core exhibited the most promising mechanical properties and desired 3D printability behavior.

Another interesting work has been reported by Alli and coworkers [66]. The group synthesized a macro-RAFT agent based on a three-arm hydroxylated star-shaped poly(3-hydroxy octanoate), (PHO). The star-shaped macro-RAFT agent was then used to synthesize polystyrene (PS) blocks, resulting in AB_3_-type PHO-*b*-PS star block copolymers. The results revealed that the kinetics of the RAFT polymerization procedure followed the free radical polymerization pattern. Moreover, it was clearly observed in the block copolymer stars that the glass transition temperature (*T*_g_) of PS, influences the plasticizing effect of the PHO soft segments.

Skandalis et al. [67] reported on the synthesis of double hydrophilic star block copolymers by RAFT polymerization, implementing the core “first” method. Ethylene glycol dimethacrylate (EGDM) was the monomer in the crosslinked core which was used as macro-CTA for the preparation of poly(2-dimethylamino ethyl methacrylate), PDMAEMA, homostar polymers. The PDMAEMA homostars were then utilized as macro-CTA for the polymerization of poly(oligo ethylene glycol)methacrylate, OEGMA, resulting in the final star block copolymers with PDMAEMA-*b*-POEGMA arms. Molar masses in the range of 23–32 kDa and molar mass dispersity in the range of 1.4–1.6 were reported. The self-assembly properties in aqueous solutions were investigated by DLS and the size of acquired nanoassemblies was ca. 44 nm. Because of the presence of the pH-responsive PDMAEMA in the structure of these star block copolymers, they hold the potential to be used effectively in nucleic acids delivery applications [68]. Sentoukas et al. [69] have prepared star-shaped copolymers with a crosslinked/hyperbranched core via the core-first method. The group utilized ethylene glycol dimethacrylate (EGDMA) bifunctional monomer and HPMA to initially prepare hyperbranched/crosslinked cores, while OEGMA was used for the extension of the arms. By modifying the CTA/crosslinker ratio, the group synthesized architectures with different numbers and lengths of arms. The star-shaped copolymers were dispersed as single-star units in dimethyl formamide (DMF), while aqueous dispersion studies showed the formation of aggregates ranging from 50 to 300 nm, depending on the structure of each star. Qu et al. [70] utilized RAFT dispersion polymerization to develop 4-arm (PNIPAAm-*b*-PS)_4_ star block copolymer nanospheres. PS was the hydrophobic core, while PNIPAAm served as the temperature-responsive corona. The synthesis of the star block copolymers was achieved by employing a tetra-functional macro-RAFT agent. The size of the nanospheres was found to be influenced by the DP of each block, increasing with increasing DP of PS and decreasing with increasing DP of PNIPAAm. When compared with linear PNIPAAm-*b*-PS block copolymers with similar compositions, the size of the spheres was found to be the same. Additionally, the LCST of the stars varied with the DP of PNIPAAm in the system. This work can be important for our better understanding of how the topology of PNIPAAm influences the temperature-responsive phase transition.

A common strategy for the preparation of complex macromolecular architectures, like star block copolymers, is the combination of different polymerization techniques to achieve the desired results. In this context, an interesting work combining RAFT polymerization and photo-initiated thiol-ene (PITE) click reaction for the preparation of star-shaped block copolymers was published by Xue et al. [71]. At first, a 4-arm allyl ether was prepared and was used for the preparation of an 8-arm allyl ether via PITE. The as-prepared 4-arm and 8-arm allyl ethers served as the cores for the synthesis of the 4-arm and 8-arm stars, respectively. The linear arms, comprising poly(2-dimethylamino ethyl methacrylate) and poly(tert-butyl acrylate) blocks, were synthesized by sequential RAFT polymerization in both possible sequences, i.e., PDMAEMA-*b*-PtBA or PtBA-*b*-PDMAEMA. The final star block copolymers were prepared by PITE click reaction. Detailed characterization of the stars showed that parameters like the number of arms, the block sequence, and the block ratio are critical for the properties of the material. Zeng et al. [72] utilized RAFT-mediated polymerization-induced self-assembly (PISA) to prepare well-defined star copolymer nano-objects and more specifically, (AB)_n_ star block copolymer nano-objects. Also, the R-RAFT-mediated PISA and the Z-RAFT-mediated PISA approaches were compared in this study. GPC results revealed, on the one hand, that the R-RAFT-mediated PISA only led to block copolymers with broad molecular weight distributions and, on the other hand, that the Z-RAFT-mediated PISA approach was more effective, leading to the preparation of well-defined star block copolymers with narrow molecular mass distributions.

## 5. Hyperbranched Copolymers

**Figure 5:**
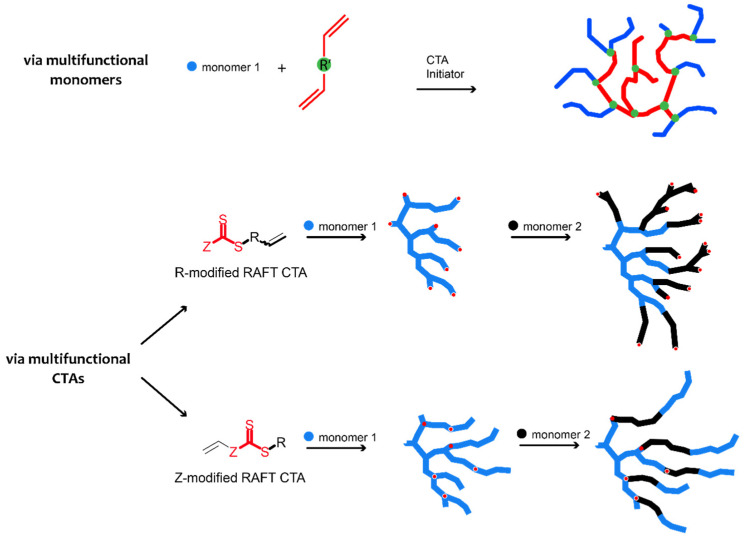
Schematic illustration of hyperbranched block copolymer synthesis starting from multifunctional monomers, and R- or a Z-modified RAFT CTA.

RAFT polymerization is a versatile tool for the synthesis of hyperbranched copolymers. Hyperbranched or highly branched copolymers are an attractive alternative to dendrimers, a class of symmetrical polymer species with sequential branches emanating from a central point. Even though the most recent reported materials exhibit monodisperse and well-defined structures, their synthesis normally requires multiple steps rendering the resulting products time intensive and considerably costly for applications [73,74,75]. Particularly, RAFT combined with advanced reaction mechanisms has managed the limitation of hyperbranched polymers’ irregularity and has enabled more efficient control over topology, composition, and molar mass dispersity (Table 1). Gelation, which used to be firmly associated with hyperbranched polymers produced by free radical copolymerization, due to uncontrollable chain length, is now easily prevented [76]. Subsequently, the research field is shifting towards the usually more convenient one-step fabrication of hyperbranched polymers (Figure 5).

A standard route to attaining hyperbranched architecture through the RAFT technique is utilizing a proper quantity of a branching agent. The compatibility of the method to various branching agents, meaning multifunctional monomers, usually referred to as crosslinkers or multifunctional chain transfer agents (CTAs), allows for numerous designs and applications [77,78]. As an example, Ai et al. [79] studied the copolymerization of butyl methacrylate (BMA), antifouling vinyl-functional Econea, and degradable divinyl-functional poly(ε-caprolactone) (PCL) in the presence of the CTA 2-cyano-2-propyl dodecyl trithiocarbonate. Divinyl PCL and Econea monomers were specifically synthesized to receive hyperbranched copolymers with antifouling properties and degradable branching bridges. A 10 to 34 mol % of PCL to the total vinyl monomer feed range was utilized, resulting in relatively low branching degrees around 0.8–3.9 mol %. Antibacterial assays indicated that the fabricated copolymer coatings presented good antibacterial activity due to controlled degradation. Several other research works investigate similar syntheses of hyperbranched copolymers with degradable bridges for marine antifouling coatings [80]. Dai et al. [81] reported on the development of hyperbranched polymers by a hydrophobic bifunctional monomer, a tertiary carboxybetaine ester acrylate possessing the antifouling group N-(2,4,6-tri chlorophenyl) maleimide (TCB-TCPM) when copolymerized with methacrylic anhydride (MAAh), a degradable divinyl monomer. Hydrolysis of TCB-TCPM releases TCPM groups to attack biofouling and zwitterionic groups to resist the adhesion of biofouling. In addition to the antifouling behavior, the effect of reaction duration on chain length and the degree of branching was investigated. Interestingly, by increasing polymerization time from 6 to 24 h, the average molecular weight of the precipitated polymer rises from 1.5 to 1.9 × 10^4^ g/mol while the branching degree remains at 6 mol %, fact that displays well-controlled growth by the RAFT process. Another study revealed that the degradation rate is dependent on the branching agent when bifunctional N,N-adipic bis(diacetone acrylamide hydrazone) (DAA_2_H), MAAh, and ethylene glycol dimethacrylate (EGDMA) were examined [82].

The copolymerization of monofunctional with bifunctional monomers to prepare promising amphiphilic hyperbranched copolymers for biomedical applications has also been explored [83,84]. For instance, Selianitis et al. [84,85] worked on the design of novel poly(oligo(ethylene glycol)methyl methacrylate)-co-poly(2-(diisopropylamine)ethyl methacrylate), P(OEGMA-co-DIPAEMA), and hyperbranched polyelectrolyte copolymers, and investigated the encapsulation of hydrophobic drug curcumin within their self-assembled structures, as well as multi-complexation behavior with short-linear DNA molecules in aqueous media. Polymerizations were carried out in 1,4-dioxane solution where a CTA to crosslinker ratio ((4-cyano-4-(phenylcarbonothioylthio) pentanoic acid to EGDMA) was kept constant at 1/1.1 and yielded high conversion copolymers with narrow molecular mass distributions and high average weights of molecular masses. A similar approach was reported by Blackburn et al. [86] for the synthesis of fluorescently labeled pH-responsive poly(2-propyl acrylic acid-co-2-(dimethylamino) ethyl methacrylate-co-disulfide diacrylate) (PAA-co-DMAEMA-co-DSDA) polymers, where redox-sensitive DSDA was the branching agent. The copolymers surprisingly displayed relatively increased PAA composition since AA is a low reactivity monomer. The authors hypothesize that further increasing the RAFT agent ratio to initiator would decrease molecular mass and cause a decrease in the molar mass dispersity value trending toward 1, due to increased controllability of the reaction. Molar mass dispersity values around 1.1 to 2.6 were observed, while conjugation of rhodamine B ethylene diamine onto the polymers was possible, meaning that the last could potentially serve as drug delivery vehicles.

Trivinyl monomers have also been explored as multifunctional branching agents. Chen and his team [87] have employed highly reactive trivinyl methyl-2-ethylidene-5-hydroxyhept-6-enoate methacrylate (MEDMA), from the substituent δ-lactone, 3-ethylidene-6-vinyltetrahydro-2H-pyran-2-one, to achieve chemoselective polymerization mediated by the CTA 2-cyanoprop-2-yl-dithiobenzoate. This can be used for the production of both linear and hyperbranched topologies with predetermined molar mass and moderate dispersity indices (<1.22), and is suitable for thiol−ene click chemistry to introduce functional groups such as amino and carboxy groups.

Except for hyperbranched copolymers with random monomer distribution along the polymer chain, as seen in the copolymers described above, several segmented highly branched copolymers have been produced [88,89]. An interesting study conducted by Bachler et al. [90] involved a comprehensive comparison between two different strategies to obtain modular segmented hyperbranched pentafluorophenyl and 2,3,5,6-tetra fluorophenyl acrylate polymers, the one being via RAFT copolymerization with a divinyl crosslinker (diethylene glycol diacrylate) and the other via RAFT self-condensing vinyl polymerization (SCVP). RAFT SCVP is another commonly applied approach for synthesizing versatile hyperbranched polymers. Typically a polymerizable group is installed into the Z(C=S)SR CTA structure on either the R-group (R group approach) or Z-group (Z group approach) side which then operates a chain transfer monomer (CTM) commonly referred to as transmer. Both routes yielded highly branched polymers with controllable branching frequency, whereas RAFT SCVP offered improved control and access to a higher branching degree since gelation was a limiting factor with the copolymerization approach when high equivalents of crosslinker were used. P. R. Calvo et al. [91] studied the synthesis of hyperbranched aminobisphosphonic acid polymers via RAFT, utilizing an acrylamide functional polymerizable transmer followed by a post-polymerization modification aiming to create degradable imine hydrogels. The acrylamide moiety of the transmer operated as the polymerizable R-group of similar reactivity to a protected amine functionalized monomers in order to achieve consistent branch incorporation and homogeneity between the length of the branches. Variations of monomer to transmer ratios, from 50:1 to 5:1 in four analogous polymerizations, revealed that reducing the monomer feed ratio led to an increase in branching as well as to a decrease in the branch length. In addition, the branching degrees calculated from NMR spectroscopy were in close agreement with the theoretical values, meaning the synthetic route displayed high control. Several other reports establish that stoichiometric ratios of monomers and transmers may be employed as a tuning factor for a targeted degree of branching and average molecular weight [92,93,94]. Moreover, the influence of the monomer structure in terms of stereochemistry has also been demonstrated by M. Nandi et al. To investigate the self-assembly of stearoyl-appended pendant hyperbranched polymers based on amino acids, for selective gelation of oil from oil–water biphasic mixtures, the researchers fabricated several L-amino acid-based methacrylate monomers, which were modified at the N terminal and bore carbon chains (-CH_3_, CH(CH_3_)_2_, CH(CH_3_)CH_2_CH_3_, CH_2_C_6_H_5_). The hyperbranched polymers obtained from RAFT SCVP indicated that the higher bulkiness of the –R chain increases branching. The bulkiness causes diffusion and decelerates the active radical propagation, resulting in a higher number of polymers with low molecular weight, which interact with the relative transmer incorporating branching points [95].

The majority of the transmers reported involve the R-group approach benefiting from the easier modification reactions of CTAs. The Z-group approach has been especially reported for hyperbranched copolymers of certain targeted functionality and morphology [89,96]. Generally, even though RAFT SCVP significantly increases polymer tunability by the use of specific transmers, the synthesis of transmers may require multiple steps, rendering this approach time consuming [97,98].

Recently, Lin and coworkers [99] reported on a methacrylate-based model system, where a series of hyperbranched copolymers of MMA and EGDMA operating as a crosslinker were produced, aiming to quantify RAFT-mediated gel point suppression in branched polymers with high crosslinker content and high targeted molecular weight. The model system describes the transition between inter- and intramolecular crosslinking by a new phenomenological parameter called the crosslinking tendency (CT), expressing the ratio of excess vinyl content to the number of primary chains per multi-vinyl monomer. By varying total vinyl concentration and the molar ratio of EGDMA to MMA, it was found that for CT < 100, excessive intermolecular crosslinking, and therefore gelation, are suppressed. Later, Forrester and his group [100] exploited this information to synthesize hyperbranched thermoplastic poly(acrylate glycerol) biopolymers to develop sustainable wood adhesives. Instead of a divinyl monomer, a bifunctional CTA, dibenzyl carbonotrithioate, was used as a branching agent, and polymerization was conducted in diluted conditions of a monomer mixture resulting from an established equilibrium of an esterification reaction of crude glycerol. CT was set as less or equal to 20, and the multi-vinyl acrylate-glycerol combination produced polymers with close to the targeted number of average molar mass (as high as 1 MDa) and relatively low molar mass dispersion, while gel point conversion of over 88% was achieved via polymerization in methanol.

Other advances involving RAFT towards this polymer architecture, include its expansion to bulk [101], surface-initiated [102,103,104] polymerization, polymerization-induced self-assembly [89,105], combination with ring-opening polymerization [106] or even flow chemistry. Specifically, using flow chemistry is a promising method for improving SCVP. Rong and his team combined photoinduced electron/energy transfer PET-RAFT and SCVP, utilizing a flow reactor to synthesize a series of hyperbranched poly(poly (ethylene glycol methyl ether) acrylates) (PPEGMEA) [107]. A comparison between batch and flow reactions indicated that utilizing a flow reactor is notably beneficial for increasing polymerization yield.

## 6. Nanogels

**Figure 6:**
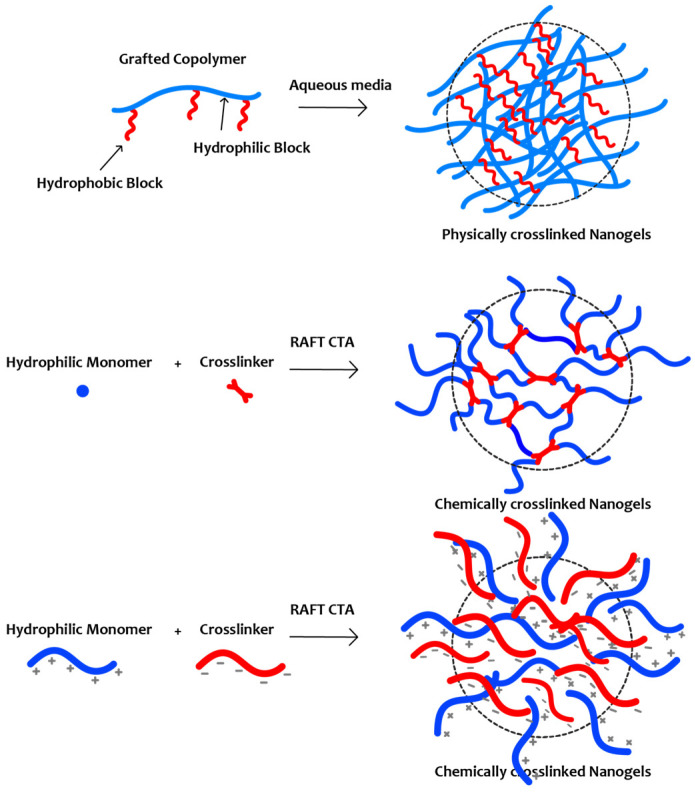
Schematic illustration of different types of formed nanogels.

In recent years, nanogels have attracted the interest of polymer scientists due to their significant properties as drug or gene delivery carriers. Nanogels are crosslinked polymeric materials, possibly having a core-shell morphology for the efficient entrapment of drugs or other bioactive compounds (Figure 6). The physical and chemical stimuli of these materials play an essential role in transferring a variety of biomolecules in vitro and in vivo. Because of their low toxicity, their sizes in the nanoscale, and their biodegradation properties, nanogels are extensively studied for the entrapment of large and small biomolecules like DNA, proteins, and chemotherapeutic and imaging agents. This section focuses on recent studies on the synthesis, characterization, and potential applications of nanogels synthesized by RAFT polymerization (Table 1).

### 6.1. Chemically Crosslinked Nanogels

Chemically crosslinked nanogels are synthesized through covalent bonds that form a permanent network, ensuring high stability and durability. Nanogels created by Shen et al. [108], utilize linear poly(ethylene glycol) (PEG) and/or nonlinear polymers with oligo(ethylene glycol) side polymeric chains, forming a stable core-shell structure. The RAFT polymerization technique enables precise control over the nanogel size, with the formed structure exhibiting significant stability against freeze–thaw cycles and maintaining stability in NaCl media for over three months, and in bovine serum albumin (BSA) solution for one week. The inclusion of nonlinear polymers enhances their stability further, indicating their robust nature for drug delivery applications where chemical stability and biocompatibility are crucial. The nonlinear nanogels were stable for four days in fetal bovine serum (FBS) solution compared to linear nanogels, which were stable for two days. Finally, these nanogels were investigated via an in vitro cytotoxicity experiment. The chemical stability of the RAFT chain transfer agents (CTAs) gives superior biocompatibility to the RAFT prepared nanogels.

Bhuchar et al. [109] constructed a thermoresponsive nanogel system with a degradable core. RAFT polymerization was used to synthesize crosslinked micelles utilizing poly(2-methacryloyloxyethyl phosphorylcholine), poly(MPC) as the hydrophilic shell chains and thermo-sensitive cores comprising poly(methoxydiethylene glycol methacrylate) (poly(MeODEGM) and poly(2-aminoethyl methacrylamide hydrochloride) (poly(AEMA). The nanogels were successfully synthesized with controllable dimensions and narrow size distributions. Moreover, these nanogels could encapsulate different biomolecules such as insulin, BSA, and β-galactosidase. The results revealed the nanogel’s dependency on the crosslinker concentration as well as the fact that the behavior of the nanogel was influenced by the presence of AEMA in the core. The release experiments for the nanogel formulations presented a gradual release of insulin for more than 48 h.

Ahmed et al. [110] developed a novel double responsive carbohydrate-based nanogel system by RAFT methodology. The nanogels consisted of poly(ethylene glycol) methyl ether methacrylate (PEGMA) as the shell, 2-lactobionamidoethyl methacrylamide (LAEMA) or 3-gluconamidopropyl methacrylamide (GAPMA) as the core and 2,2-dimethacroyloxy-1-ethoxypropane (CL) as the crosslinker. The synthesized nanogels were capable of encapsulating and co-delivering plasmid DNA and proteins. The sizes of these thermo- and pH-responsive nanogels were in the range of 15–20 nm at 37 °C, as measured by dynamic light scattering (DLS). The temperature-responsive nature of the nanogels permits the efficient entrapment of biomolecules and, at acidic conditions, allows the release of bioactive compounds in endosomes. In vitro experiments were performed in order to evaluate the toxicity of nanogels. The results show the dependence of cytotoxicity on the concentration of the crosslinker. Furthermore, the nanogels containing LAEMA and the largest crosslinker concentration showed the high loading capacity of the biomolecules, high gene expression in Hep G2 cells, and low cytotoxicity.

Van Driessche and co-workers [111] synthesized a new pH-responsive nanogel system based on amphiphilic block copolymers. The formed nanogels consisted of poly(N, N-dimethylacrylamide-b-pentafluorophenylacrylate), PDMA-b-PFPA diblock copolymer synthesized via RAFT polymerization. DLS measurements were used to determine the average diameter (ca. 90 nm) of the formed nanogels. Doxorubicin (DOX) was used as the hydrophobic drug for encapsulation in the core of the nanogels. The drug-loaded nanogels showed a burst release under acidic conditions. In vivo experiments on tumor-bearing zebrafish embryos demonstrated a remarkably decreased systemic toxicity but improved tumor accumulation and tumor growth reduction.

A series of photocleavable nanogels designed and synthesized by RAFT polymerization were developed by Xin and co-workers [112]. Specifically, they synthesized a hydrophilic photocleavable nanogel based on methoxy polyethylene glycol methacrylate (MPEGMA) and UV-light responsive crosslinker 5-(acryloyloxy)-2-nitrobenzyl acrylate (ONB). Coumarin 102 was used as the hydrophobic tracer encapsulated in the hydrophobic core of the nanogels. DLS measurements were performed to determine the size and size-dispersity indices of the empty and the drug-loaded nanogels. The encapsulation efficiency and drug loading were 24.1% and 4.29%, respectively. Furthermore, in vitro cytotoxicity studies of these nanogels revealed remarkable cell viability, making them potential candidates for drug delivery applications.

### 6.2. Nanogels Formed by Hydrophobic Interactions

Nanogels formed through hydrophobic interactions involve the physical self-assembly of amphiphilic copolymers. The work by Don and co-workers [113] showcases the synthesis of poly(acrylic acid-b-N-Isopropylacrylamide) diblock copolymers via RAFT polymerization, which form nanogels upon self-assembly in aqueous media. These nanogels respond to various stimuli including pH, temperature, and enzymes, demonstrating their potential in drug delivery, particularly in enhancing the intracellular accumulation of drug-loaded nanogels in cancer cells. The amphiphilic nature of these nanogels contributes to their dynamic assembly and disassembly, allowing for controlled drug release. Indeed, the formed cationic nanogels were able to pass through the cell membrane and improved the intracellular accumulation of DOX-loaded nanogels in MCF-7/ADR breast cancer cells. Moreover, the cationic nanogels were capable of encapsulating the Rose Bengal photosensitizer, triggering remarkable damage in the cancer cells under irradiation. The acquired results revealed the potential use of these nanogels as drug vehicles.

An exciting study regarding thermoresponsive nanogels via RAFT polymerization was described by Stefanello et al. [114]. They used hyaluronic acid (HA) derivatives which were modified with a biocompatible poly[di(ethylene glycol) methacrylate-co-oligo(ethylene glycol) methacrylate] poly(DEGMA-co-OEGMA) thermoresponsive statistical copolymer to form nanogels. The formed HA-p-poly(DEGMA-co-OEGMA) nanogels could self-assemble into spherical gel nanoparticles with a radius of about 150 to 214 nm above 37 °C. A two-photon dye was encapsulated in the hydrophobic component of the nanogel and transferred to macrophages in vitro. The results showed that the HA-p-poly(DEGMA-co-OEGMA) nanogels could successfully deliver hydrophobic dye to macrophages of hepatic and splenic tissues.

Another interesting study regarding synthesizing novel biocompatible and thermoresponsive nanogels was reported by Kolouchova and co-workers [115]. The synthesis of these nanogels is based on a hydrophilic poly[N-(2-hydroxypropyl) methacrylamide] (PHPMA) or poly(2-methyl-2-oxazoline) (PMeOx) block and a thermosensitive poly[N(2,2-difluoroethyl) acrylamide] (PDFEA) block. RAFT polymerization was used to synthesize these amphiphilic thermoresponsive nanogels. The thermoresponsive character of PDFEA gave the possibility to control nanogels self-assembly upon heating in aqueous media. The ratio between the hydrophilic and the hydrophobic block was an essential parameter for forming the most suitable nanogel in terms of size. These nanogels could encapsulate an adequate concentration of magnetically active fluorine atoms for magnetic resonance imaging (^19^F MRI) purposes. In vitro MRI experiments exhibited excellent responsiveness of the diblock copolymer agents, and the formed nanogels were non-cytotoxic for different cell lines.

### 6.3. Nanogels Formed by Complexation or Coacervation

Recent advances have highlighted the formation of nanogels through complexation or coacervation, particularly focusing on polyion complex micelles (PIC) and surfactant-polyion complex micelles (SPIC). These nanogels are formed through the electrostatic interaction between oppositely charged polymers or between polymers and surfactants, leading to a non-covalent, reversible nanoscale network. Such structures are highly responsive to environmental changes, making them suitable for targeted drug delivery. Peng et al. [116] synthesized nanogels consisting of RAFT-synthesized statistical poly(N-vinylpyrrolidone-co-N-vinylformamide) copolymers, which utilize a redox-responsive crosslinker, covalently bonded with DOX, for the formation of prodrug nanogels. In vitro DOX release profiles revealed that by increasing the crosslinking density at pH 7.4, a reduction in DOX release occurred. Also, the DOX-loaded nanogels presented an antitumor effect on HeLa cells. The intracellular release profile exhibits a controllable character by adjusting the crosslinking density of the nanogels. Such structures exhibit controlled drug release triggered by changes in the redox state, demonstrating the potential of complexation-based strategies in responsive therapeutic delivery systems.

**Table 1:** Overview of copolymer structures discussed in this review.

Polymer Name	Macromolecular Architecture	RAFT Agent	Synthetic Method	Citation
P((MMA-statBTPEMA)-block-PDMAm)-graft-PDMAm	Graft copolymer	CDTPA and BTPEMA	PET-RAFT	Corrigan et al. [44]
P(EGMA-co-BTPEMA)-b-PS-g-PS	Graft copolymer	BTPEMA and CEPA	PET-RAFT	Yang et al. [45]
P((OEGMA-co-BTPEMA)-b-NIPAAm)-g-PNIPAAm	Graft copolymer	CPDTC and BTPEMA	PET-RAFT	Xu et al. [46]
PMAA-g-PPC	Graft copolymer	CPAD	RAFT	Alagi et al. [47]
PHEMA-g-PCL	Graft copolymer	CPADB	RAFT and ROP	Guo et al. [48]
POEGMA-b-PMMA)-g-P(GMA-N_3_)	Graft copolymer	CPADB	RAFT	Thankappan et al. [49]
PEHA or PDMA-g-EtOx	Bottlebrush- and comb-like copolymers	BCTPA	RAFT and ROP	Kim et al. [50]
(cellulose-4-dimethylaminopyridine) -g-P(AA-co-MMA)	Graft copolymer	Bagasse Cellulose-modified CTA	RAFT and click chemistry	Assen et al. [53]
Dextran-g-PHPMA	Graft copolymer	Dextran- modified CTA	RAFT and post-polymerization functionalization	Ikkene et al. [32,54,55]
Dextran-g-PHPMA	Graft copolymer	Dextran- modified CTA	RAFT and post-polymerization functionalization	Ferji et al. [56]
CS-g-PNIPAAm and Hep-g-PNIPAAm	Graft copolymer	DDMAT	RAFT	Pilipenko et al. [57,58]
(PS-b-PI)_arms_-(DVB-co-PS)_core_	Star copolymer	CDTP	RAFT	Ge et al. [64]
(PMMA-b-TMSPMA)_arms_-(Peptides)	Star copolymer	CDB	RAFT	Volski et al. [65]
PS_arms_-PHO_core_	Star copolymer	PHO-based CTA	RAFT	Alli et al. [66]
(PDMAEMA-b-POEGMA)_arms_-EGDMA_core_	Star copolymer	CPAD	RAFT	Skandalis et al. [67]
POEGMA_arms_-(PHPMA-co-EGDMA)_core_	Star copolymer	CPAD	RAFT	Sentoukas et al. [69]
(PNIPAAm)_arms_-(PS)_core_	Star copolymer	TTC	RAFT	Qu et al. [70]
(PtBA-b-PDMAEMA)-(allyl-ether)_core_	Star copolymer		RAFT and click chemistry	Xue et al. [71]
PDMA_arms_	Star copolymer	TTC	RAFT PISA	Zeng et al. [72]
BMA, Econea and Divinyl PCL	Hyperbranched copolymer	CDTP	RAFT	Ai et al. [79]
mod-tCBEA-co-MAAh	Hyperbranched copolymer	CPDT	RAFT	Dai et al. [81]
DAAH-co-MAAh-co-EGDMA	Hyperbranched copolymer	CPDT	RAFT	Pan et al. [82]
POEGMA-co-DIPAEMA	Hyperbranched copolymer	CPAD	RAFT	Selianitis et al. [84]
P(AA-co-DMAEMA-co DSDA)	Hyperbranched copolymer	CDCTPA	RAFT	Blackburn et al. [86]
mod-MEDMA	Hyperbranched copolymer	CPDBA	RAFT	Chen et al. [87]
PPFPA, PTFPA	Hyperbranched copolymer	Modified CTA	SCVP RAFT	Bachler et al. [90]
P(AbPA)	Hyperbranched copolymer	Modified CTA	SCVP RAFT	Calvo et al. [91]
P(SAA)	Hyperbranched copolymer	Modified CTA	SCVP RAFT	Nandi et al. [95]
P(MMA-co-EGDMA)	Hyperbranched copolymer	CDB	RAFT	Lin et al. [99]
P(AG)	Hyperbranched copolymers	DBCNT	RAFT	Forrester et al. [100]
Linear PEG and nonlinear POEG	Nanogel	TTC and CPAD	RAFT	Shen et al. [108]
P(MPC)-PAEMA-PMeODEGMA)	Nanogel	Macro-MPC	RAFT	Bhuchar et al. [109]
POEGMA-(LAEMA-co-CL), POEGMA-(GAPMA-co-CL)	Nanogel	CPAD	RAFT	Ahmed et al. [110]
HA-P(DEGMA-co-OEGMA)	Nanogel	CPAD	RAFT	Stefanello et al. [114]
PAA-b-PNIPAAm	Nanogel	DMP	RAFT	Don et al. [113]
PHPMA-PDFEA, PMeOx-PDFEA	Nanogel	CPAD and CDTP	RAFT	Kolouchova et al. [115]
PDMA-b-PFPA	Nanogel	PABTC	RAFT	Van Driessche et al. [111]
P(VPD-VF)	Nanogel	MECTP	RAFT	Peng et al. [116]
MPEGMA-co-ONB	Nanogel	CPDB	RAFT	Xin et al. [112]

## 7. Potential Applications of Branched (Co)polymers

**Figure 7:**
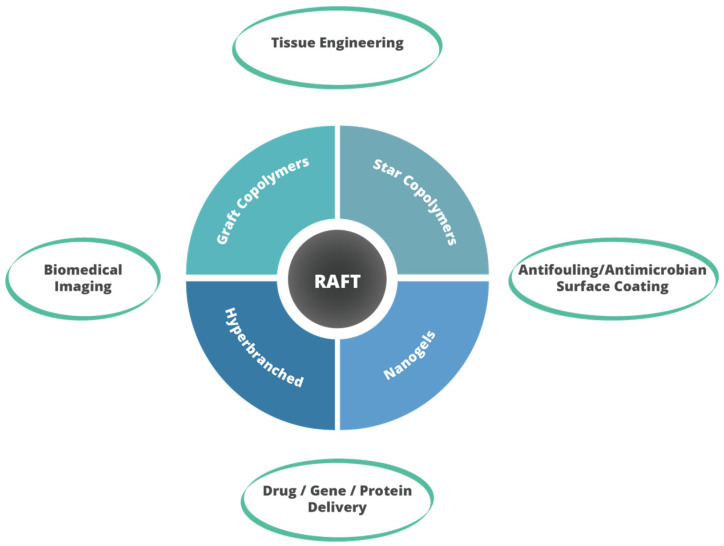
Potential applications of branched copolymers.

Copolymers synthesized through RAFT polymerization, such as branched copolymers, star-shaped polymers, hyperbranched polymers, and nanogels, exhibit a broad spectrum of applications across various fields (Figure 7), notably in the biomedical sector. The synthesis techniques and structural versatility offered by RAFT polymerization enable the creation of polymers with specific functionalities, architectures, and responsive behaviors, making them ideal for targeted applications.

The increased loading capacity and the presence of numerous functional groups in branched copolymers make them suitable carriers for therapeutic molecules, including drugs and genes. Their amphiphilic nature allows for efficient encapsulation and release mechanisms. The diversity in branching and functionalization can be exploited to design scaffolds that support cell growth and tissue regeneration, offering tunable degradation rates and appropriate mechanical properties.

The unique architecture of star-shaped polymers improves solubility and reduces viscosity, making them beneficial for pharmaceutical formulations where these properties are critical. The mechanical properties and degradation rates of star polymers can be tailored for use as inks in 3D printing, producing scaffolds that support cell growth and tissue regeneration.

Hyperbranched polymers with antifouling/antimicrobial properties can be applied in surface coatings to prevent protein or bacterial accumulation. Their ability to form self-assembled structures makes them suitable for encapsulating hydrophobic drugs, or bioimaging agents, while their multi-complexation behavior with biomolecules like DNA or protein offers avenues in gene/protein delivery.

The core-shell morphology and stimuli-responsive nature of nanogels make them excellent candidates for the targeted delivery and controlled release of therapeutic agents, including small-molecule drugs, proteins, and nucleic acids. The ability to encapsulate imaging agents and control their release makes nanogels useful in biomedical imaging, enhancing the contrast and specificity of diagnostic imaging techniques.

The versatility in synthesis and the potential for functionalization allow these polymers to be customized for specific applications, particularly in the biomedical field where their unique properties can be leveraged to address specific challenges in drug delivery, tissue engineering, and diagnostic imaging. The ongoing advances in RAFT polymerization techniques and polymer engineering are expected to further expand the applications of these polymers, offering innovative solutions in healthcare and beyond.

## 8. Challenges and Limitations

Despite the versatility of RAFT polymerization, the challenges and limitations associated with the synthesis and application of polymers extend beyond the control of branching degrees and molecular weight distribution.

### 8.1. Synthesis Complexity and Scalability

RAFT polymerization often involves complex synthesis steps, particularly for creating advanced architectures like star-shaped or hyperbranched polymers. These steps can include the careful design of chain transfer agents (CTAs) and monomers, precise control over reaction conditions, and potentially, multiple stages of polymerization. The complexity of these synthesis processes can lead to scalability issues, making it challenging to produce large quantities of polymers for commercial or industrial applications. Developing more streamlined synthesis protocols or adopting continuous flow chemistry approaches could help enhance scalability.

### 8.2. Cost-Effectiveness

The need for specialized CTAs, high-purity monomers, and sometimes extensive purification processes can make RAFT polymerization more expensive compared to traditional polymerization methods. Efforts to synthesize more cost-effective CTAs or to recycle them from the polymerization medium could improve the cost-effectiveness of RAFT polymerization processes.

### 8.3. Environmental and Safety Concerns

Many RAFT polymerizations are conducted in organic solvents, raising concerns about environmental impact and safety. Research into water-based RAFT polymerization or the development of greener solvents and solvent-free processes can mitigate these issues. The presence of residual monomers and CTAs in the final polymer product can pose toxicity risks, particularly for biomedical applications. Developing more efficient polymerization strategies and purification techniques to minimize these residuals is crucial.

### 8.4. Polymerization Control and Predictability

While RAFT allows for control over molecular weight and molecular weight distribution, achieving precise control over polymer microstructure (such as branching density in hyperbranched polymers) remains challenging. Advanced modeling and real-time monitoring techniques could enhance predictability and control. Although RAFT is versatile, there are limitations regarding the range of monomers that can be polymerized, especially for more polar or sensitive monomers. Expanding the range of compatible monomers through the development of novel CTAs and optimized polymerization conditions is an area of ongoing research.

### 8.5. Biocompatibility and Biodegradation

For biomedical applications, ensuring the biocompatibility of RAFT-synthesized polymers is paramount. This includes not only the polymers themselves but also any residual CTAs or crosslinkers. Designing polymers with predictable and controllable biodegradation profiles is crucial for many applications, especially in drug delivery and tissue engineering. Research into novel biodegradable linkages that can be incorporated via RAFT polymerization is necessary.

Addressing these challenges requires a multidisciplinary approach, combining advances in chemistry, materials science, and engineering. Continuous innovations in RAFT polymerization techniques, coupled with a deeper understanding of the structure–property relationships of polymers, are key to overcoming these limitations and expanding the application potential of these versatile materials.

## 9. Conclusions and Outlook

The outlook for RAFT polymerization and its derived polymers is incredibly promising, with significant potential for innovation and expansion into new applications. As we look ahead, several key areas are poised to benefit from the advances in RAFT polymerization technologies, especially in the field of branched polymers.

The versatility and precision of RAFT polymerization in synthesizing polymers with specific functionalities and macromolecular topologies make it an invaluable tool in the biomedical field. Future developments are expected to focus on creating more sophisticated drug delivery systems that can target specific tissues or cells with high precision, enhancing the efficacy of treatments while minimizing side effects. Additionally, the design of biocompatible and biodegradable polymers for tissue engineering scaffolds will advance regenerative medicine, enabling the repair or replacement of damaged tissues and organs.

As global awareness of environmental sustainability grows, the development of greener and more eco-friendly polymer synthesis methods becomes increasingly important. RAFT polymerization can contribute to this area by enabling the synthesis of polymers from renewable resources, reducing the reliance on petroleum-based monomers. Furthermore, advancements in RAFT polymerization could lead to more efficient recycling processes for plastics and other polymeric materials, contributing to a circular economy.

The control over polymer architecture that RAFT polymerization offers opens the opportunity to designing smart and advanced polymers that respond to environmental stimuli (such as temperature, pH, or light). These materials could have applications in self-healing coatings, responsive drug delivery systems, and adaptive materials capable of changing their properties in response to external conditions, offering innovative solutions for a wide range of industries.

While the potential applications for RAFT polymerization are vast, several challenges remain, including scalability, cost, and the environmental impact of polymer production. Addressing these challenges will require continuous innovation in polymer science and engineering. For instance, developing more efficient and less costly RAFT agents, exploring water-based polymerization processes, and enhancing the recyclability of RAFT polymers are critical areas for future research.

The future of RAFT polymerization will likely be shaped by interdisciplinary research that combines chemistry, materials science, biology, and engineering. Collaborations between academia and industry will be essential to translate laboratory-scale innovations into practical applications. Furthermore, leveraging advancements in computational modeling and machine learning could accelerate the design and optimization of RAFT polymerization processes and polymer properties.

In conclusion, RAFT polymerization stands at the forefront of polymer science, offering unparalleled control over polymer architecture and functionality. It carries the potential to address some of the most pressing challenges in healthcare, sustainability, and materials science. As we continue to explore and expand the boundaries of what is possible with RAFT polymerization, we can anticipate the emergence of new materials that will shape the future of technology and society.

## Data Availability

No new data were created.

## Glossary—Abbreviations

AEMA	2-AminoethylMethacrylamideHydrochloride
AG	AcrylateGlycerol
ATRP	Atom Transfer RadicalPolymerization
BDB	BenzylDithiobenzoate
BMA	ButylMethacrylate
BSA	BovineSerumAlbumin
BzTC	BenzylTrithiocarbonate
CL	22-Dimethacroyloxy-1-Ethoxypropane
DAA2H	NN-AdipicBis(DiacetoneAcrylamideHydrazone)
DMA	N,N-Dimethylacrylamide
DOX	Doxorubicin
DSDA	DisulfideDiacrylate
DVB	Divinylbenzene
ECT	EthylCyanovalericTrithiocarbonate
EGDMA	EthyleneGlycolDimethacrylate
EHA	2-EthylhexylAcrylate
EGMA	(EthyleneGlycol)MethylEtherMethacrylate
FBS	Fetal Bovine Serum
GAPMA	3-GluconamidopropylMethacrylamide
HA	Hyaluronic Acid
IPrTC	IsopropylTrithiocarbonate
LAEMA	2-LactobionamidoethylMethacrylamide
MAAh	MethacrylicAnhydride
MEDMA	Methyl-2-Ethylidene-5-Hydroxyhept-6-EnoateMethacrylate
MPEGMA	MethoxyPolyethyleneGlycolMethacrylate
MPC	MethacryloyloxyethylPhosphorylcholine
NMP	Nitroxide-MediatedPolymerization
OEGMA	Oligo(EthyleneGlycol)Methacrylate
ONB	5-(Acryloyloxy)-2-NitrobenzylAcrylate
PDFEA	Poly(N(22-Difluoroethyl)Acrylamide)
PDMAEMA	Poly(2-DimethylaminoEthylMethacrylate)
PDMAm	Poly(N,N-Dimethylacrylamide)
PEG	Poly(EthyleneGlycol)
PEGMA	Poly(EthyleneGlycol)MethylEtherMethacrylate
PFPA	Pentafluorophenylacrylate
PHO	Poly(3-HydroxyOctanoate)
PHPMA	Poly(N-(2-Hydroxypropyl)Methacrylamide)
PISA	Polymerization-Induced Self-Assembly
PMeOx	Poly(2-Methyl-2-Oxazoline)
PMMA	Poly(MethylMethacrylate)
PMAA	Poly(MethacrylicAcid)
PNIPAAm	Poly(N-Isopropylacrylamide)
PTFPA	2356-TetraFluorophenylAcrylate
RAFT	Reversible Addition-Fragmentation chain Transfer
RDRP	Reversible Deactivation Radical Polymerization
ROP	Ring-Opening Polymerization
SAA	Stearoyl-Appended Pendant Amino Acid
